# Satellite Remote Sensing of Alpine Vegetation Dynamics: Challenges and Perspectives

**DOI:** 10.1111/gcb.70968

**Published:** 2026-06-16

**Authors:** Arthur Bayle

**Affiliations:** ^1^ Univ. Grenoble Alpes, Univ. Savoie Mont Blanc, CNRS, LECA Grenoble France; ^2^ Department F.‐A. Forel for Environmental and Aquatic Sciences University of Geneva Geneva Switzerland; ^3^ Climate Change Impacts and Risks in the Anthropocene (C‐CIA), Institute for Environmental Sciences University of Geneva Geneva Switzerland

**Keywords:** alpine ecosystems, attribution, bias, greening, Landsat, mountains, reproducible workflows

## Abstract

Satellite greening has become a key tool for monitoring alpine vegetation change, but a positive vegetation‐index trend is not an ecological observation in itself. This perspective shows that interpreting alpine greening requires addressing two sequential challenges: methodological complexity, which can bias trends during image processing, and phenomenological complexity, because different ecological processes can produce similar spectral signals. Progress now depends less on producing more greening maps than on linking robust satellite trends to ground‐based ecological processes.

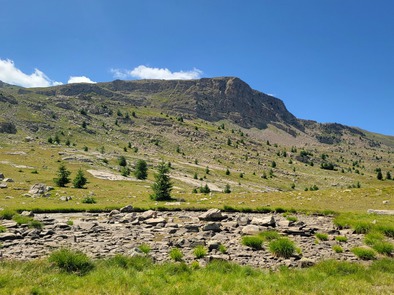

## 1 Introduction

Temperate alpine ecosystems—here broadly defined as landscapes above the treeline where seasonal snow strongly constrains growing seasons and habitat structure—are rapidly changing in the face of global change. In many mountain regions, warming is amplified relative to adjacent lowlands (Dumont et al. 2025; Pepin et al. 2025), and land‐use abandonment is prominent (Anselmetto et al. 2024; Daskalova and Kamp 2023), triggering responses across multiple compartments of the mountain system. While transformations are evident and palpable in the cryosphere—most notably through glacier retreat and reduced snow cover (Bosson et al. 2023; Matiu et al. 2021)—widespread changes in alpine plant communities are also underway (Kullman 2010). At their lower elevational limit, open alpine landscapes are contracting as forests and shrubs advance upslope (Kullman and Öberg 2025; Nicoud et al. 2025), while grasslands show increasing greenness, and thermophilous species are colonizing high‐elevation terrain (Elumeeva et al. 2013; Gottfried et al. 2012). Understanding where and how these changes unfold is essential because they reshape alpine biodiversity and ecosystem functioning, with cascading consequences for carbon and water cycling and downstream ecosystem services (Hagedorn et al. 2019; Immerzeel et al. 2020; Lamprecht et al. 2018; Theodoridis et al. 2025).

**FIGURE 1:**
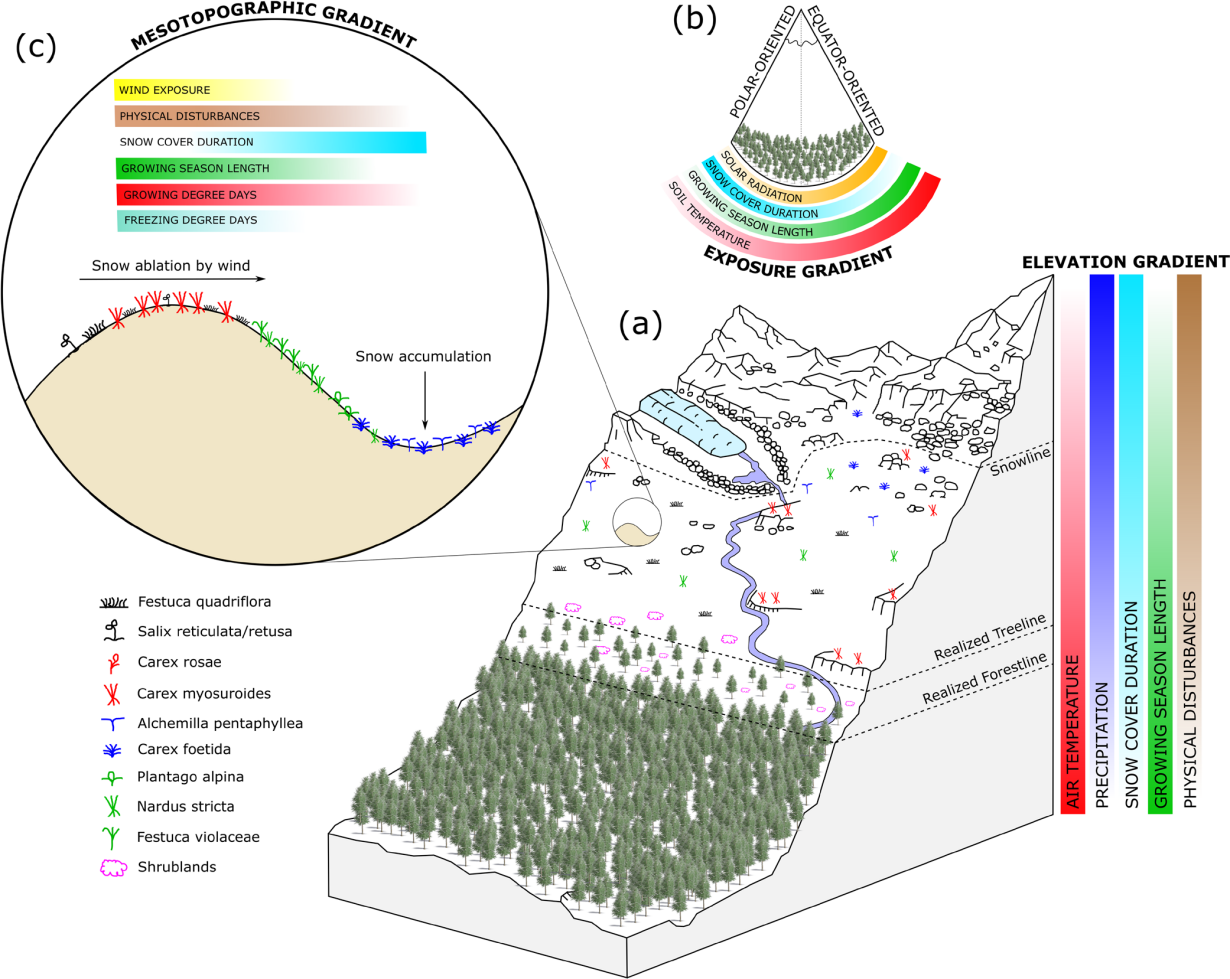
Conceptual illustration of how broad elevational constraints and fine‐scale terrain heterogeneity interact to structure alpine vegetation between the climatic treeline and the snowline. (a) Along elevation gradient, rapid declines in temperature and growing‐season energy shorten the season and intensify stress, while cryospheric influence increases toward the upper alpine where persistent snow and frost action restrict establishment to sheltered microsites. (b) Aspect further partitions this template by altering radiation and snow melting dynamics. Together these interacting gradients generate persistent patchworks of microhabitats (e.g., fellfields, grasslands, heaths, wet meadows, snowbeds, krummholz), emphasizing that alpine vegetation is not a continuous elevational belt but a mosaic whose spatial structure underpins biodiversity and mediates ecosystem responses to environmental change. (c) Superimposed mesotopographic features (ridges, slopes, hollows, benches) redistribute snow and modulate wind exposure, creating sharp contrasts in insulation, soil moisture, and growing‐season timing over tens of meters—for example, wind‐exposed ridges that remain snow‐free and frost‐prone versus snow‐accumulating depressions with delayed melt but protected soils.

Temperate alpine ecosystems have a long tradition of ecological study (Grabherr et al. 2000; Thuiller et al. 2024), and a great deal is known about their floristics, functional strategies, and environmental controls (De Frenne et al. 2013; Körner 2021). Yet they remain particularly challenging systems in which to quantify vegetation dynamics because alpine vegetation is structured by the superposition of steep elevational gradients with strong terrain controls operating at much finer scales (Choler 2018). Elevation and aspect structure macroclimatic gradients, but meso‐ and microtopography govern snow redistribution, wind exposure, radiation, soil moisture, freeze–thaw cycles, and disturbance over tens of meters, producing a fine‐grained mosaic of environmental conditions in which contrasting plant communities can occur in close proximity (Figure 1). This mosaic structure means that vegetation change is often spatially patterned rather than uniform (Bueno De Mesquita et al. 2018), complicating extrapolation from sparse observations or experiments (Scherrer et al. 2011). The result is that observed vegetation change reflects interacting drivers operating across nested spatial scales (Suding et al. 2015), making it difficult to capture representatively with traditional approaches alone. Field surveys and permanent plots provide irreplaceable ecological detail, but they are inevitably sparse relative to the spatial grain of alpine mosaics and the extent of mountain ranges (Steinbauer et al. 2018).

These limitations have motivated increasing use of satellite remote sensing, which offers repeated, spatially exhaustive observations across large areas and multi‐decadal periods. In parallel to what has been developed in the Arctic greening literature (Myers‐Smith et al. 2020), much of this work relies on long‐term trends in optical vegetation indices (VIs), commonly summarized as “greening”—that is, a positive temporal trend in VIs (and “browning” for negative trends). Yet, alpine ecosystems pose specific challenges for satellite‐based inference of vegetation dynamics. With the rapid expansion of alpine greening studies over the recent years (Anderson et al. 2020; Choler et al. 2025; Leng et al. 2026; Rumpf et al. 2022), a synthesis is needed to clarify what satellite “greening” can (and cannot) be taken to mean in alpine landscapes, to identify the main sources of uncertainty in trend estimation, and to outline a defensible path from radiometric trends to ecological processes that can serve as a common ground. This perspective paper argues that translating radiometric time series into greening trends, and translating those trends into vegetation dynamics, is constrained by two intertwined layers of complexity—(i) methodological complexity reflects that greening trends are the endpoint of a radiometric observation and processing chain into which uncertainty propagates, imprinting artefacts on observed trends, and (ii) phenomenological complexity reflects that greening trends are equifinal—different ecological phenomena can produce similar changes in greenness metrics—making process attribution ambiguous without additional constraints. Only once these two complexities are resolved sequentially—by first establishing methodologically robust trends and then constraining their ecological interpretation through process attribution—does it become defensible to use greening patterns to generalize broad‐scale vegetation dynamics and to evaluate climatic and land‐use drivers. The remainder of this paper is organized around these two complexities: I first outline how greening trends are constructed and review, non‐exhaustively, what satellites have revealed about alpine vegetation change, motivate Landsat as the multi‐decadal backbone archive, and then develop methodological and phenomenological complexities before proposing a stepwise framework from robust trend estimation to process attribution and, only then, driver inference. Finally, I discuss how progress in the coming decade may depend on combining complementary observation systems across scales with workflows that remain transparent, reproducible, and broadly accessible, so that increasingly diverse satellite observations can be connected more effectively to ecological processes.

## 2 Measuring Vegetation Dynamics from Satellite Remote Sensing

Quantifying vegetation dynamics depends on measurement frameworks that sample ecological change across space and time. In its broadest sense, measurement assigns numerical values to phenomena according to consistent rules (Stevens 1946; Voje et al. 2023), and any observation is defined by its spatial resolution and extent, and its temporal interval and duration (Estes et al. 2018). Historically, vegetation change was documented through field‐based surveys and long‐term monitoring (Davis et al. 2005; Luken 1990) within a hypothesis‐driven paradigm designed to test specific predictions (Hari et al. 1983). Such observations provide high ecological detail but remain limited in spatial and temporal coverage. The proliferation of large, publicly available datasets—satellite remote sensing being a prominent example—has accelerated a shift toward data‐driven analyses, where patterns are first extracted from heterogeneous observations and only later interpreted ecologically. This shift expands coverage and consistency, but it also propagates uncertainty because processing and modeling choices can introduce error and bias at multiple stages. The rapid uptake of satellite remote sensing in alpine vegetation studies mirrors this transition.

Optical satellite remote sensing offers repeated, synoptic observations of the Earth's surface across multiple spectral bands, enabling vegetation to be monitored consistently over vast extents and across long time spans, typically ranging from years to decades (Pettorelli et al. 2014). At the sensor level, satellites record radiance; after radiometric calibration and atmospheric correction, these observations are commonly expressed as surface reflectance products at the pixel scale. The spectral component of vegetation is then summarized through parametric transformations of reflectance known as vegetation indices (VIs). Rather than measuring vegetation properties directly, VIs act as spectral proxies that are often related to canopy greenness, vegetation cover, or photosynthetic activity, depending on ecosystem context and background conditions. Common examples include the normalized difference vegetation index (NDVI; Rouse et al. 1974), the enhanced vegetation index 2 (EVI2; Huete et al. 2002; Jiang et al. 2008), the near‐infrared reflectance of vegetation (NIRv; Badgley et al. 2017), or the Kernel NDVI (kNDVI; Camps‐Valls et al. 2021). Different vegetation indices emphasize different dimensions of vegetation dynamics and differ in their sensitivity to artefacts (Glenn et al. 2008; Wang et al. 2023; Zeng et al. 2022). Broad red–NIR indices such as NDVI and EVI2 are widely used to monitor structural greenness and vegetation dynamics, but NDVI is generally more sensitive to soil background variations and saturates earlier in dense canopies, whereas EVI2 was designed to reduce atmospheric influences and retain sensitivity in denser vegetation. NIRv has been developed to reduce soil background effects and shows strong correlation with vegetation photosynthesis (Badgley et al. 2019), while kNDVI has been proposed as a more sensitive alternative to NDVI for tracking terrestrial carbon dynamics (Wang et al. 2023). Vegetation‐index choice is therefore not neutral in greening studies, because it influences both the robustness of trend estimates and the ecological interpretation of the resulting signal. In practice, alpine vegetation dynamics still rely predominantly on NDVI as a general indicator of land‐surface greenness, which has the advantage of allowing broad patterns of vegetation state to be described consistently without requiring overly specific ecological interpretation at the outset.

**FIGURE 2:**
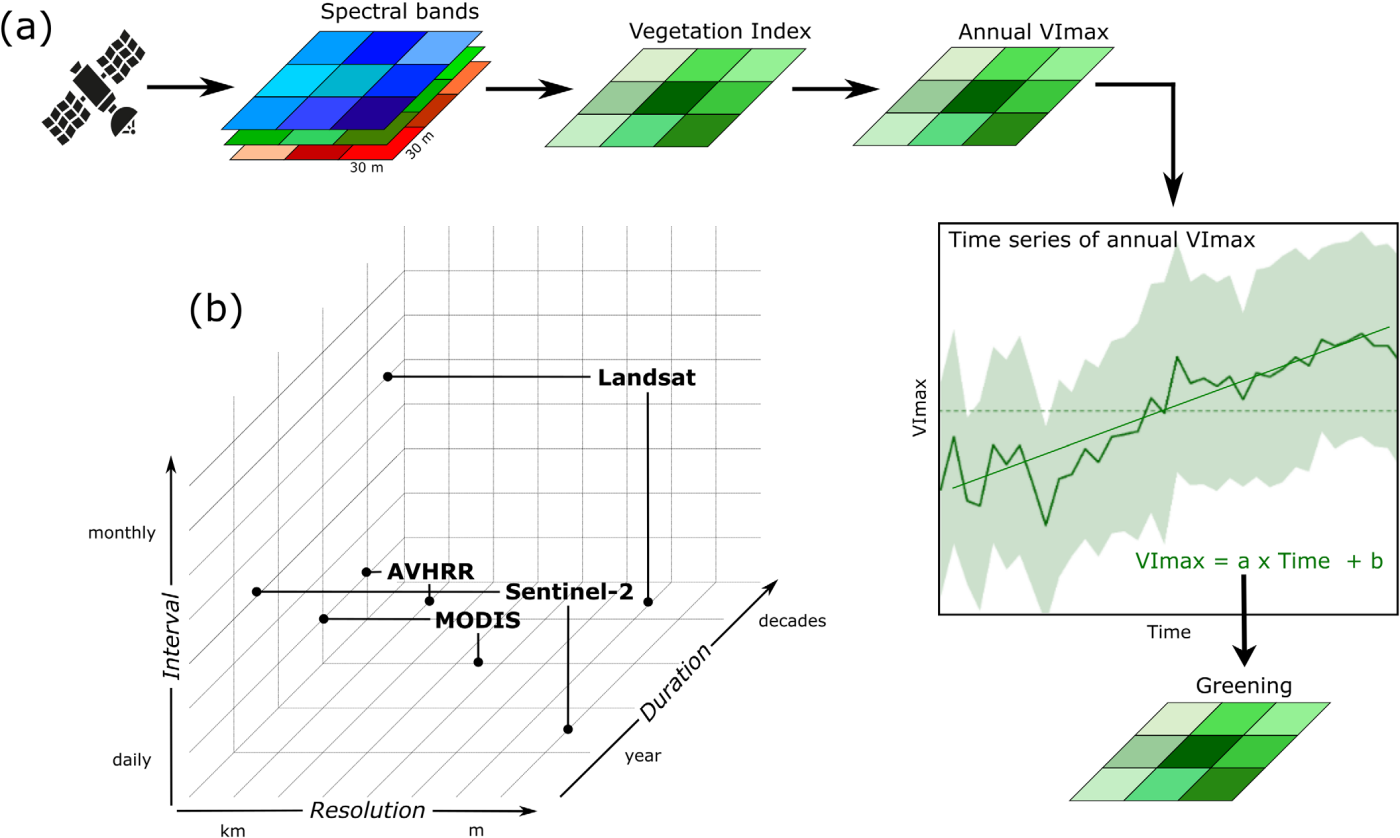
Spatial and temporal analysis of alpine vegetation from satellite data. (a) Chain of processing from satellite remote sensing measuring of Earth's surface reflectance to the obtention of pixelwise greening trends (b) Interval, resolution and duration of the four constellations used the most for computation of greening trends. The “extent” parameter is not represented here as they all provide global coverage.

Translating VI imagery into “vegetation dynamics” requires converting irregular image acquisitions into consistent time series (Figure 2a). This typically involves a sequence of practical steps: (i) quality filtering and masking of contaminated observations (clouds, cloud shadows, and often snow), (ii) defining the temporal window of interest (e.g., peak growing season, fixed calendar periods, or a snow‐free season), and (iii) temporal aggregation to obtain a single metric per interval (e.g., monthly means, seasonal medians, seasonal maxima, or integrals). Alternative approaches fit seasonal curves to estimate phenology metrics (e.g., onset, peak timing, season length) and reduce sensitivity to missing observations (Berner et al. 2020). Within this framework, long‐term change is commonly formalized as a trend in an aggregated VI metric. Trends are often estimated using trend models (most commonly linear slopes), sometimes with robust estimators that reduce sensitivity to outliers (e.g., Theil‐Sen estimators), and increasingly with models that account for temporal autocorrelation (Ives et al. 2021; Lewińska et al. 2023). Change is then summarized by its magnitude (e.g., VI units per year or decade, or relative change) and its direction (positive versus negative). Many studies also report statistical significance of trends; however, interpretation of “significant greening” for pixels treated as independent has been questioned in the remote sensing literature, mostly because spatial dependence and multiple testing can violate the assumptions underlying pixel‐wise tests (Ives et al. 2021). In contexts where vegetation is sparse, greening is also occasionally defined as the expansion of vegetated area—defined by the trespassing of a VI threshold—over time (Anderson et al. 2020; Roland et al. 2024). Another option is to quantify change using time‐integrated VI (or related area‐under‐the‐curve metrics), which may better capture cumulative seasonal vegetation activity than peak VI alone and has been used as a surrogate for aboveground productivity in cold ecosystems (Tassone et al. 2024; Yan et al. 2022). However, such metrics require dense and regular observations to characterize the seasonal trajectory reliably, making them more readily applicable to AVHRR‐ or MODIS‐type archives than to the more irregular Landsat record.

Indeed, greening measurements depend fundamentally on the satellite archive used (Figure 2b), reflecting trade‐offs among spatial detail, revisit frequency, and record length. Sentinel‐2 offers high spatial resolution (10–20 m) and frequent revisits (~5 days with two satellites), making it well suited for mapping fine‐scale patterns and within‐season dynamics, but its record begins in 2015 and is therefore limited for multi‐decadal trend assessment. At the other extreme, AVHRR provides near‐daily observations over more than four decades, but at coarse spatial resolution (~1 km), which can obscure fine‐grained ecological patterns in heterogeneous landscapes (Ju and Masek 2016). MODIS occupies an intermediate position in time and space, with near‐daily observations since 2000 at moderate spatial resolution (typically 250–500 m for vegetation products), enabling consistent regional‐to‐continental analyses but still averaging over fine‐scale habitat mosaics. Landsat sits between MODIS and Sentinel in spatial detail while offering a longer record than MODIS, combining multi‐decadal continuity with moderate spatial resolution (~30 m). However, its nominal revisit frequency (16 days per satellite) and frequent data loss to clouds, shadows, and snow create irregular temporal sampling—limitations that are particularly acute in mountain environments.

Selecting an appropriate sensor, metric, VI, and processing strategy is therefore a matter of aligning the observation design with the ecological phenomenon of interest—while acknowledging that the resulting greening trend reflects a chain of decisions about filtering, seasonal definition, aggregation, and trend modeling. In ecosystems with strong spatial heterogeneity and pronounced seasonality, these decisions can meaningfully shape the patterns that emerge from analysis. For that reason, a key prerequisite for interpreting reported greening signals is clarity about how trends were constructed (which index, seasonal window, compositing rule, trend model, and quality filtering) and how sensitive conclusions are to those choices. These sensitivities motivate the need to separate trend robustness from ecological interpretation—issues that are developed in the methodological and phenomenological complexity sections.

## 3 What Satellites have Revealed About Alpine Vegetation Dynamics

Global alpine ecosystems are increasingly being studied using satellite imagery. Work now spans numerous mountain systems, including mountain regions across Europe (Alonso‐González et al. 2024; Carlson et al. 2017; Rumpf et al. 2022; Spracklen and Spracklen 2025; Theodoridis et al. 2025), the Andes (Lepage et al. 2023; Polk et al. 2020), New Zealand (Hua et al. 2021), Central and North American mountains (Correa‐Díaz et al. 2021; Emmett et al. 2019; Moore et al. 2025; Potter 2019), and High Mountain Asia (Anderson et al. 2020; Gouda and Dubey 2026; Leng et al. 2026; Macek et al. 2025), covering a wide range of bioclimatic alpine conditions (Testolin et al. 2020). Across these studies, NDVI remains by far the dominant vegetation index, most often derived from Landsat, and more occasionally from MODIS or AVHRR. In contrast to high‐latitude systems where browning has been reported alongside warming (Berner et al. 2020), a recurring finding across satellite studies is that alpine “greening” is widespread and often dominant. As a striking example, Zou et al. (2025) reported that 99% of global alpine zones were classified as experiencing significant greening over the last four decades, raising the question of whether per‐pixel hypothesis testing remains meaningful under high statistical power and massive multiple comparisons. Regional studies across multiple ranges echo this overall dominance of positive trends, including in the European Alps, where Rumpf et al. (2022) found greening across 77% of the area over a comparable multi‐decadal period, while Choler et al. (2021) reported 55% of above‐forest habitats exhibiting significant greening since 2000. Importantly, if attention is shifted from hypothesis testing (“significant” vs. “non‐significant”) to the sign of estimated trends, the picture becomes even more one‐sided: the distribution of slopes is overwhelmingly positive, implying that most of the remaining area is not browning but simply exhibits weaker, noisier, or less detectable positive change. In all three studies, browning occupies only a very small fraction of the landscape (typically ≤ 1%) and is largely confined to localized disturbance footprints rather than pervasive declines. However, studies conducted toward the dry end of alpine climates show a less uniform greening signal: the balance between greening and browning appears to shift along hydroclimatic gradients and through time, with browning becoming more likely where warming and reduced snow cover exacerbate moisture stress (Leng et al. 2026; Liu et al. 2021; Macek et al. 2025; Moore et al. 2025).

These broad‐scale patterns are consistent with the strong topographic modulation of greening reported within mountain ranges, where aspect and snow persistence create sharp gradients in energy and water limitation. Across regions, aspect effects are also evident at broad scales, with polar‐facing slopes gaining greenness faster than equator‐facing slopes (Tian and Tian 2026; Yin et al. 2023; Zou et al. 2025), supporting the role of thermal constraints being lifted under climate change (Keenan and Riley 2018). The European Alps provide a well‐documented illustration of this topoclimatic structuring: using MODIS, Choler et al. (2021) mapped greening across the Alpine arc and showed that trends were far from uniform, with strong greening concentrated in specific elevation bands and exposures, frequently on north‐facing (polar‐facing) and relatively sparsely vegetated slopes. Extending analyses back to the early 1980s and at finer spatial resolution, Rumpf et al. (2022) reported broadly consistent spatial structuring (e.g., stronger greening on north‐facing slopes). Finally, Choler et al. (2025) identified that these north‐exposed and sparsely vegetated greening hotspots were mostly composed of snowbeds that responded to earlier snowmelt, suggesting that in more humid alpine ecosystems, warming and earlier snowmelt can benefit plant growth. Moore et al. (2025) suggested opposite patterns in the more arid southern Rockies. Locally, warming and reduced snow cover were associated with browning trends, consistent with increasing moisture limitation (Maurer et al. 2020). Taken together, these findings support a relaxation of thermal limitation in many snow‐dominated mountains, alongside a progressive shift toward more water‐limited systems in others (Berg et al. 2017; Keenan and Riley 2018). These studies show that satellite archives now allow alpine vegetation responses to be compared consistently across mountain ranges and across steep topoclimatic and hydroclimatic gradients, revealing both widespread greening and emerging contexts where greening slows—or locally reverses—under increasing moisture limitation.

In contrast to the rapid progress in mapping greening patterns, fewer studies have directly linked satellite‐derived greening to specific ecological dynamics such as changes in composition, growth forms, phenology or vegetation structure. In many cases, the ecological meaning of a VI trend is left implicit or inferred indirectly, and direct validation remains comparatively rare. Nonetheless, a small but growing set of studies has begun to bridge this gap. On mountain summits, Landsat‐based greening has been associated with increasing richness of thermophilous, low‐stature species, either directly (Dentant et al. 2024) or indirectly (Spracklen and Spracklen 2025), and threshold‐based approaches have been used to infer expansion of subnival vegetation into previously sparsely vegetated terrain (Anderson et al. 2020). Greening in recently deglaciated areas has also been studied and associated with primary succession of herbaceous species in the European Alps (Bayle et al. 2023; Knoflach et al. 2021) and in Peru (Polk et al. 2020). Near the forest–alpine ecotone, several studies suggest that greening can coincide with woody dynamics (Arekhi et al. 2018; Baglioni et al. 2025; Corimanya et al. 2025). Airborne laser scanning showed strongest greening near the ecotone but could not uniquely separate forest expansion from changes in low‐stature vegetation (Bolton et al. 2018), whereas combining Landsat trends with tree‐level photointerpretation provided more direct evidence that a substantial share of greening can coincide with increasing tree cover in parts of the European Alps (Bayle et al. 2025). Even though they did not directly attribute greening to a specific ecological process, Theodoridis et al. (2025) strongly emphasized the contribution of woody expansion in the southern Balkan mountain ranges. By comparison, despite repeated ground evidence for shrub expansion in mountain environments (De Toma et al. 2025; Formica et al. 2014; Grigoriev et al. 2022), satellite‐based attribution of greening to shrub encroachment has been less common than in Arctic systems (Nill et al. 2022). One of the first large‐scale attempts in a temperate mountain range is Turtureanu et al. (2025), who reported in the Carpathians that greening patterns were more consistent with shrub encroachment (Ericaceae and Juniperus) than with treeline shifts.

## 4 Landsat as the Backbone Archive for Alpine Vegetation Dynamics

**FIGURE 3:**
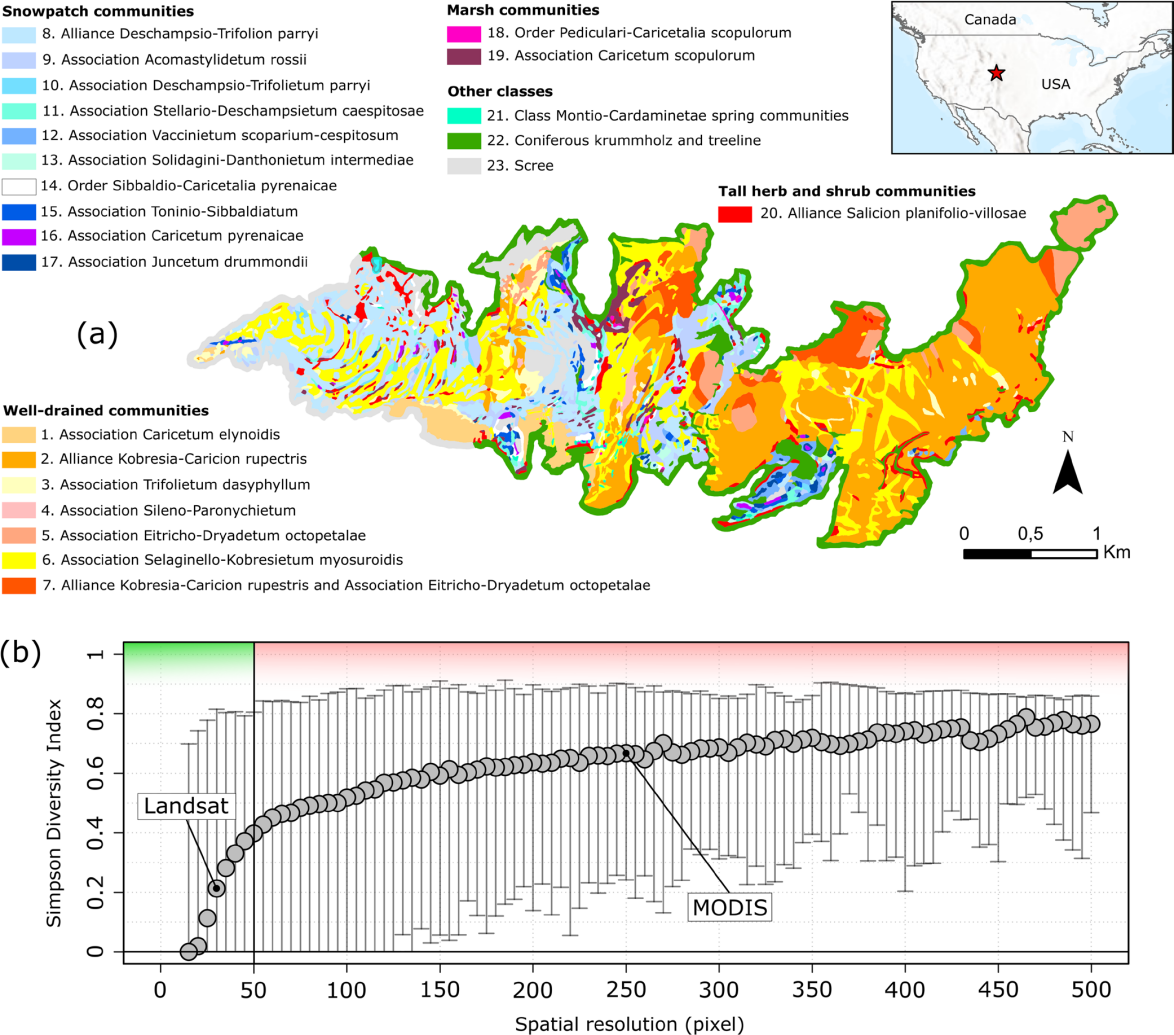
Spatial patterns and scale dependence of alpine vegetation diversity at Niwot Ridge. (a) Alpine vegetation map of Niwot Ridge (Colorado, USA) with initial classes numbered from 1 to 23. Bold titles indicate the level of aggregation at which the Simpson Diversity Index has been computed. (b) Simpson Diversity Index of alpine plant communities computed for grids with spatial resolution ranging from 15 × 15 m to 500 × 500 m. Spatial resolution of Landsat and MODIS satellites is shown. An inflection point near 50 × 50 m is shown by a vertical line. Map lines delineate study areas and do not necessarily depict accepted national boundaries.

Detecting and interpreting alpine vegetation dynamics requires reconciling two fundamental observational needs: (i) sufficient spatial detail to meaningfully represent alpine habitat mosaics (Figure 1), and (ii) sufficient temporal duration to separate directional change from strong interannual variability and to capture multi‐decadal environmental forcing. This second point is especially relevant because a large fraction of observed global warming has occurred since the late 1970s, with warming rates increasing relative to earlier 20th‐century periods. Satellite constellations differ markedly in the trade‐offs they offer between spatial resolution, revisit frequency, and record length (Figure 2). While the spatial heterogeneity of alpine habitats is widely acknowledged (Billings 1973; Choler 2018), the spatial resolution required to capture habitat‐specific response is elusive (Dedieu et al. 2016; Kollert et al. 2026). As an illustrative experiment, a detailed vegetation map of alpine plant communities from Niwot Ridge (Colorado, USA) (Komárková and Webber 1978) was used to test for intra‐pixel heterogeneity along a gradient of spatial resolution (Figure 3a). First, highly detailed plant communities were aggregated into ecologically meaningful habitat classes. Then habitat heterogeneity was quantified using the Simpson Diversity Index, computed pixel‐wise across emulated grids from 15 × 15 m (approaching Landsat scale and finer) to 500 × 500 m (coarse, MODIS‐like). The resulting pattern is strongly scale dependent. At coarse resolutions (≈500–100 m), SDI values remain high (roughly 0.8–0.5) because single pixels necessarily combine multiple vegetation communities. As resolution improves—particularly below ~50 m—SDI declines steeply, with the median approaching 0 at 15 m, indicating that pixels increasingly align with the grain of the habitat mosaic and become dominated by single community types (Figure 3b). This pattern is consistent with studies showing improved discrimination of alpine plant communities when using medium‐ rather than coarse‐resolution satellite imagery (Dedieu et al. 2016). However, this result should be interpreted as an illustration rather than a general calibration of the spatial grain of alpine mosaics. The Niwot Ridge map reflects a particular combination of climatic regime (cold, snow‐dominated temperate alpine), biogeographic context (Nearctic alpine flora), and mountain topography characteristic of the Front Range, all of which shape the size, arrangement, and mixing of vegetation patches. In other mountain systems, the grain of alpine mosaics may differ substantially so the resolution threshold at which pixels become dominated by single habitat types may shift accordingly (Leng et al. 2024). Applying the same scaling analysis to comparable vegetation maps from other alpine regions would therefore be a valuable next step for assessing how generally the Niwot Ridge pattern holds, specifically along hydroclimatic gradients (Malfasi and Cannone 2021; Mendes et al. 2025). Even with these caveats, this example illustrates why the moderate‐resolution Landsat archives occupy a crucial middle ground for alpine studies: they provide sufficient spatial detail to retain key mosaic structure while also offering the multi‐decadal continuity required for robust trend analysis.

Yet, Landsat remains a compromise rather than an ideal observation system. The Landsat program was initiated in the early 1970s to provide systematic, repeat imaging of the land surface and has since become the longest‐running civilian Earth‐observation effort (Loveland et al. 2022). For most vegetation time‐series applications, what is meant by the “Landsat constellation” is the 30 m multispectral lineage that begins in the mid‐1980s with Landsat 5 Thematic Mapper (TM) and continues through Landsat 7 ETM+, Landsat 8 OLI, and Landsat 9 OLI‐2 (hereafter referred to as TM, ETM+, OLI, and OLI2). The archive is not seamless: mission transitions, evolving acquisition strategies, and occasional technical disruptions have left a time series that is continuous in principle but uneven in practice. Throughout its existence, and increasingly since the shift to open‐access data, substantial effort has been devoted to harmonizing Landsat reflectance observations through time and across sensors (Berner et al. 2023; Roy, Kovalskyy, et al. 2016; Roy, Zhang, et al. 2016; Zhang and Roy 2016). A second, closely related feature of the Landsat archive is that the number of usable observations per pixel has not been constant throughout time (Zhang et al. 2022). In principle, each Landsat sensor provides a 16‐day revisit cycle, but the effective observation density depends on how many satellites are operating concurrently, on acquisition policies, and on mission‐specific disruptions. In the mid‐1980s to late‐1990s, most analyses rely primarily on Landsat 5 TM, when temporal sampling was constrained by a single‐sensor revisit and by periods of reduced acquisition and downlink capacity. The launch of Landsat 7 ETM+ in 1999 created a dual‐satellite era with an ~8‐day nominal repeat, increasing the probability of capturing snow‐free, cloud‐free windows. This period was partially offset after 2003 by the ETM+ Scan Line Corrector (SLC) failure, which introduced persistent data gaps within scenes, although the mission continued to contribute valuable coverage through long‐term acquisition planning. Following the end of Landsat 5 in 2012 and the launch of Landsat 8 OLI in 2013, observation density increased again as the archive entered a new sensor generation with sustained global acquisition. The launch of Landsat 9 OLI‐2 in 2021 marked the first period in the Landsat era when two modern sensors operated concurrently without major scene‐wide technical failures, establishing a new baseline of more regular 8‐day sampling (Masek et al. 2020). Landsat therefore provides unmatched temporal depth at moderate resolution, but its non‐uniform sampling through time and across missions is an inherent constraint on trend‐based interpretations of vegetation dynamics.

## 5 Complexity I—Methodological Complexities

Greening is a numerical construction that starts from top‐of‐atmosphere radiance and ends with trends computed from aggregated vegetation indices. Throughout this processing chain, uncertainty accumulates and propagates, progressively decoupling the observed signal from ground‐based change. Resolving methodological complexity therefore means maximizing the ground‐related component of the signal. Broadly, uncertainty has two components: (i) uncertainty and bias affecting the reflectance measurements themselves, and (ii) uncertainty introduced by how reflectance observations are filtered, aggregated, and modeled to derive trends. This distinction applies regardless of the satellite constellation used; however, the remainder of this section focuses on Landsat given its importance for alpine vegetation dynamics (Figure 3).

Landsat reflectance estimates are sensitive to multiple sources of uncertainties. A baseline component is residual uncertainty from instrumental noise or imperfect atmospheric correction, estimated between 3% and 7% for TM, ETM+ and OLI (Chander et al. 2009; Markham et al. 2014; Markham and Helder 2012). In addition, instruments aboard Landsat satellites have different spectral response functions and overall characteristics. TM and ETM+ surface reflectance products have been evaluated as broadly consistent (Claverie et al. 2015), although inconsistencies have been reported in the Arctic (Berner et al. 2020). By contrast, ETM+ and OLI are showing larger discrepancies, resulting in systematically higher OLI NDVI compared to ETM+ NDVI (Roy, Kovalskyy, et al. 2016), which could artificially inflate greening trends (Berner et al. 2020). Generic approaches have been proposed to calibrate surface reflectance across sensors and reduce discrepancies (Berner et al. 2023; Choler et al. 2025; Roy, Kovalskyy, et al. 2016). Beyond sensor characteristics, Landsat acquisition geometry has also varied over time because orbit maintenance and mean local overpass time have not been perfectly constant within and across missions (Qiu et al. 2021; Roy et al. 2020). Because the radiance recorded by an optical sensor depends on sun–surface–sensor geometry, shifts in local overpass time translate into changes in solar zenith and azimuth angles at acquisition, even when surface properties are unchanged (Gao et al. 2014; Nagol et al. 2015). Zhang and Roy (2016) quantified the effect of Landsat 5 orbit drift on NDVI and modeled a spurious browning trend of ~0.0006 NDVI year^−1^, corresponding to an apparent decrease of ~0.016 NDVI over the Landsat 5 record, attributable solely to changes in acquisition geometry. Roy, Kovalskyy, et al. (2016) and Roy, Zhang, et al. (2016) proposed the c‐factor correction to normalize the Landsat time series to constant geometries (Zhang et al. 2016). In mountains, topography introduces additional radiometric uncertainty. Slope and aspect control illumination conditions and therefore influence reflectance (Riano et al. 2003), creating spatial inconsistencies. In principle, changes in sun and sensor geometry could also modify how these topographic effects manifest throughout the Landsat archive, and corrections have been proposed (Sola et al. 2016). However, evaluations suggest that applying topographic correction does not necessarily improve temporal consistency (Qiu et al. 2018). Finally, because Landsat acquisitions have tended to occur later in the morning in more recent periods, cast‐shadow distribution can change systematically through time, with pixels transitioning from a shaded to a shadowless state, a potential, still poorly explored source of bias in mountain areas (but see Choler et al. (2025)). Importantly, these uncertainties are not expected to affect all vegetation indices equally, as different VI formulations are subject to atmospheric effects, BRDF, topographic illumination, and background contamination at different levels of sensitivity (Zeng et al. 2022). The choice of VI should therefore be considered explicitly when assessing methodological robustness. In combination, these factors can bias greening estimates upward or downward, complicating ecological interpretation. At minimum, studies should make known bias mechanisms explicit and demonstrate that key conclusions are not contingent on uncorrected effects.

A second component of methodological complexity arises from how vegetation indices are aggregated before trend computation, and in particular from how aggregation strategies handle irregular data acquisition. In greening workflows, seasonal reflectance is often summarized using maxima or high quantiles of VI (VImax) before trend computation (Francini et al. 2023). Yet, this annual maximum is a transient phenomenon (Hwang and Mallat 1992), whose estimates increase asymptotically with the number of observations (Berner et al. 2020). In other words, with more clear‐sky observations, the probability of capturing annual peak greenness increases (Berner et al. 2023). This issue is compounded when vegetation phenology shifts through time, because a trend in annual peak VI may then reflect not only changes in peak vegetation state, but also changes in the timing of that peak and the probability of sampling it. This bias can occur for any constellation, but it is particularly critical for Landsat because observation density has increased through the archive (Zhang et al. 2022), producing systematically higher VImax estimates in recent years than in early years and thereby inflating greening trends (Bayle et al. 2024). For alpine ecosystems, consequences are amplified because growing‐season length and cloudiness both vary sharply along environmental gradients, producing strong fine‐scale spatial gradients in the number of usable clear‐sky observations (Hu et al. 2019). Bayle et al. (2024) modeled that, above the treeline in the European Alps, artificial greening trends could reach up to 0.001 NDVI year^−1^, representing around 30% of the observed greening trends. Because the bias magnitude differs among habitats, comparisons of “which community is greening most” can be blurred unless sampling effects are explicitly accounted for. Importantly, the same sampling bias may also reduce the detectability of browning, causing genuine negative trends to be diluted into weak greening or apparent stability, and thereby potentially contributing to the overwhelming dominance of greening reported in some large‐scale studies (Zou et al. 2025). Corrections have been proposed in the literature with phenological modeling (Berner et al. 2023) or avoidance strategies—relying only on annual estimates based on sufficient observations (Dentant et al. 2024; Macek et al. 2025; Raynolds et al. 2013)—being the most recurrent in the literature. A complementary pathway is to improve cloud detection in mountains, because standard masks can systematically over‐classify cold, clear pixels as clouds when temperature‐based assumptions break down along steep elevation gradients (Qiu et al. 2017). At minimum, observation counts and their temporal evolution should be reported systematically, and robustness to compositing choices and influential years should be demonstrated. Together, these measurement‐ and aggregation‐level effects can generate spatially structured artefacts that mimic habitat‐specific greening or browning, making methodological robustness a prerequisite for any ecological attribution.

## 6 Complexity II—Phenomenological Complexities

Once methodological sources of bias have been minimized and a greening trend can be treated as robust, it provides strong evidence that change has occurred on the ground, but it does not specify what has changed ecologically. This motivates the central phenomenological complexity—equifinality: different ecological phenomena can ultimately produce similar greening trends. Alpine ecosystems are currently undergoing rapid vegetation change across multiple growth forms, from herbaceous communities (Gottfried et al. 2012; Steinbauer et al. 2018) to shrubs (Cannone et al. 2022; Francon et al. 2021) and trees (Anselmetto et al. 2024; Camarero et al. 2015; Nicoud et al. 2025; Tasser et al. 2007). Although one might expect larger VI shifts for major transitions in vegetation structure and cover (e.g., grassland to forest) than for within‐community species turnover, such relationships are not systematic in alpine landscapes. This is because greening magnitude is inherently relative and strongly conditioned by the initial surface state, so similar ecological changes can yield different VI responses—and vice versa. This ambiguity is further reinforced by vegetation‐index choice, because different VIs emphasize different dimensions of vegetation dynamics and may therefore highlight different aspects of the same underlying ecological change (Glenn et al. 2008; Wang et al. 2022). Moreover, greening trends may reflect not only structural or compositional vegetation change, but also shifts in the phenology, physiological activity, or functional traits of otherwise compositionally unchanged vegetation, phenomenon extensively reported in global ecosystems (Piao et al. 2019). In alpine ecosystems, earlier green‐up, delayed senescence, longer snow‐free growing seasons, or increase in biomass can all alter the magnitude of peak VI metrics without necessarily implying changes in vegetation composition or structure (Choler 2023; Möhl et al. 2022). For instance, earlier snowmelt may both advance green‐up and increase the time available for heat accumulation, either of which may raise peak VI without requiring compositional change. At the same time, these phenological shifts may also occur together with changes in vegetation composition, making the ecological meaning of the trend more ambiguous. As a result, a positive greening trend may arise from different combinations of structural, phenological, and physiological processes, each shaped by the tight interaction of climate and topography across space and time in alpine ecosystems. Resolving this phenomenological complexity therefore requires attributing greening to the most plausible underlying ecological process, which generally demands constraints beyond the trend itself.

Attribution approaches can be grouped into two categories: (1) inference‐based attribution, in which local context—such as position in the habitat mosaic or along climatic and topographic gradients, and more broadly environmental conditions such as the timing of snowmelt—is often mobilized to narrow interpretations and infer the ecological process (Carlson et al. 2017; Choler et al. 2021, 2025; Rumpf et al. 2022; Turtureanu et al. 2025). For instance, when high‐magnitude greening is concentrated along the forest–alpine ecotone, it may be consistent with a contribution from woody expansion and an upward shift in treeline position (Bolton et al. 2018). Conversely, when greening is concentrated in open alpine grasslands far from ecotones, it may be more consistent with phenological changes, increased biomass or productivity within an otherwise similar community than with a shift in life form or vegetation structure (Choler 2015). Yet, such inferences should be treated as hypothesis rather than conclusions. (2) direct attribution, in which spectral trends are linked to ecological change using independent information—such as diachronic aerial or very‐high‐resolution imagery, plot resurveys, long‐term monitoring, repeated field measurements (e.g., species composition, cover, biomass), or dendrochronology—used to document local on‐the‐ground change and link it to spectral trajectories. This remains an ideal scenario, because such ancillary data rarely approaches the spatial coverage and temporal depth of satellite archives. Phenomenological complexity thus lies less in detecting where greenness changes than in determining which ecological change that spectral shift actually represents.

An alternative but promising approach is to work backwards from process to pattern, thereby inverting the equifinality problem. Greening is convenient because it reduces vegetation dynamics to a single dimension, potentially discarding information available in other spectral bands. By identifying ecological components a priori, spectral unmixing methods can be mobilized to derive trends in specific components of the ground surface. For instance, Lewińska et al. (2023) provided a compelling example by deriving time series of ground‐cover fractions (green vegetation, non‐photosynthetic vegetation, and soil) in Eurasian grasslands, allowing change to be partitioned into interpretable components that would be difficult to diagnose from a single greenness index. In alpine environments, however, this strategy is less straightforward. At the scale of plant communities, the assumption of stable and homogeneous endmembers at Landsat spatial resolution is difficult to maintain, both because alpine ecosystems are dominated by mixed pixels and because the spectral properties of a given community can vary strongly across its environmental niche (Schweiger et al. 2017). Owing to topographic control, such variability can emerge over very short distances, so that intra‐class spectral variability may be large even locally, introducing substantial uncertainty into model estimation (Zhang et al. 2019). This also makes the construction of representative calibration datasets particularly demanding, since they must capture variability across snow regimes, substrates, moisture conditions, exposure, and vegetation states (Hoad et al. 2025). A more realistic alternative in alpine landscapes may therefore be to move from endmember‐based unmixing toward proportion‐based learning. Rather than seeking stable pure spectra for each plant community, finer‐resolution imagery or detailed vegetation maps could be used to estimate the composition of Landsat pixels over calibration areas, and these mixed proportions could then be related directly to Landsat spectra through supervised learning. This would not remove the need for extensive calibration data, but it would shift the problem from identifying idealized pure endmembers—often unrealistic in alpine mosaics—to quantifying the composition of mixed pixels over representative training sites (Immitzer et al. 2018; Metzler and Sader 2005), thereby offering a more realistic path toward process‐resolving observables than greenness alone.

## 7 From Radiometric Trends to Ecological Processes: A Way Forward

**FIGURE 4:**
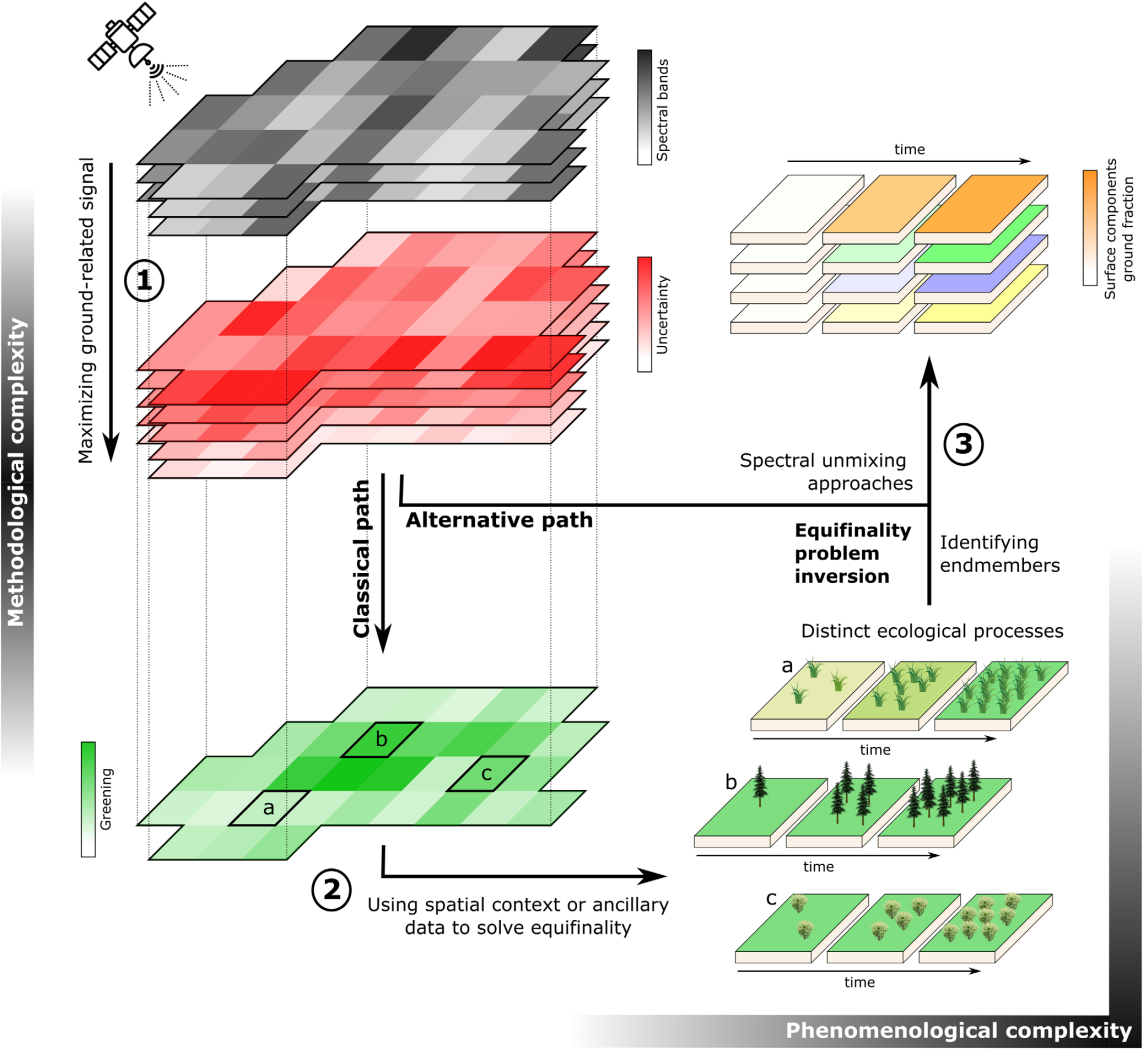
Conceptual framework for interpreting satellite‐derived alpine greening. (1) Methodological complexity summarizes how processing choices and observation uncertainties can affect radiometric time series, and how bias correction aims to produce more robust spectral reflectance and greening trends. Phenomenological complexity shows how robust spectral trends can be translated into ecological processes using (2) inference‐based or direct attribution or alternatively (3) the equifinality problem inversion through spectral unmixing approaches.

Satellite greening maps have become a default output for assessing alpine vegetation change, but the preceding sections argue that their value depends on how uncertainty is handled and how the signal is translated into ecological processes. A greening trend is not an ecological observation; it is an endpoint of radiometric measurement and processing choices, and it is ecologically ambiguous because multiple processes can produce similar trajectories. The implication is not that alpine greening studies are unreliable, but that robust interpretation should follow a sequential logic (Figure 4).

First, establish trend robustness by minimizing methodological artefacts, thereby maximizing the fraction of the signal that is plausibly ground‐related (Section 5). As a minimum standard for reproducibility and cross‐study comparability, studies should report the full processing chain used to derive greening trends, including: (1) the sensor(s) and products used; (2) data source and preprocessing workflow; (3) atmospheric correction workflow, cross‐sensor harmonization, and any BRDF normalization applied; (4) the VI used and why it was chosen; (5) the compositing rule, including seasonal window and summary metric; (6) the trend model used; and (7) the statistical testing framework, including the null hypothesis and any treatment of spatial dependence or multiple testing. Regarding methodological biases, Table 1 summarizes the main sources of uncertainty and proposes both a minimum and best‐practice response for each, together with key references. Whether treated minimally or ideally, potential sources of bias should be stated explicitly, and any omitted corrections should be justified in light of the study objective and computational constraints. For example, BRDF harmonization can be computationally demanding to apply consistently across multi‐decadal, large‐area stacks and may be omitted in practice; in that case, authors should justify the omission and argue that it would not alter the qualitative ecological interpretation. More generally, the expected direction of any uncorrected bias—whether toward inflation or deflation of greening trends—should be stated explicitly, together with its likely implications for ecological interpretation. For example, failing to account for temporal changes in observation counts or for cross‐calibration issues in the Landsat archive can both inflate apparent greening trends (Bayle et al. 2024; Berner et al. 2023). Reducing uncertainty through careful corrections and transparent reporting is crucial to establish a shared methodological baseline, enabling meaningful comparisons across study sites and regions.

**TABLE 1:** Key sources of methodological bias in alpine greening analyses and recommended responses, from minimum acceptable treatment to best practice, to help establish a shared methodological baseline across studies.

Source of methodological bias	Minimum acceptable treatment	Best‐practice treatment	Key references
Cross‐sensor calibration	Explicitly state that cross‐sensor harmonization was not applied; justify omission and discuss expected direction of bias	Apply published cross‐sensor harmonization/calibration procedures consistently across the archive, or derive local coefficients	Claverie et al. (2015), Roy, Kovalskyy, et al. (2016), Roy, Zhang, et al. (2016), Berner et al. (2023), and Choler et al. (2025)
Acquisition geometry/BRDF effect	Report acquisition geometry variables and state that BRDF normalization was not applied; justify why it is unlikely to alter qualitative conclusions	Apply BRDF harmonization to normalize reflectance to a common viewing/illumination geometry	Gao et al. (2014), Nagol et al. (2015), Roy, Kovalskyy, et al. (2016), Roy, Zhang, et al. (2016), and Zhang and Roy (2016)
Topographic illumination effects	Acknowledge that slope and aspect affect reflectance and discuss whether this may bias temporal comparisons, especially across exposures	Test and apply topographic correction where it improves temporal consistency; evaluate correction performance rather than assuming improvement	Riano et al. (2003), Sola et al. (2016), and Qiu et al. (2018)
Cast‐shadow dynamics through time	Identify whether systematic changes in shadow distribution are plausible in the study area and discuss their likely effect on trend direction	Mask or explicitly model cast‐shadow effects where feasible, especially in steep terrain and long archives	Choler et al. (2025)
Cloud/snow masking errors in mountains	Report the masking procedure used and acknowledge potential over‐masking or under‐masking in steep, cold terrain	Improve cloud/snow screening using mountain‐adapted approaches and test sensitivity of trends to masking choices	Qiu et al. (2017)
Uneven observation density through time	Report observation counts per pixel (or at least by habitat/elevation band) and their temporal evolution; justify omission and discuss expected direction of bias	Restrict trend estimation to years or pixels with sufficient observations, or explicitly correct for sampling effects using phenological modeling	Berner et al. (2020), Berner et al. (2023), Bayle et al. (2024), and Choler et al. (2025)

Second, constrain ecological interpretation through process attribution using ancillary data, whether via inference, direct attribution, or alternative unmixing approaches (Section 6). Where direct attribution is feasible, satellite trajectories should be evaluated against independent observations spanning the same period (e.g., plot resurveys, repeat photography or aerial imagery, dendrochronology). At minimum, studies should acknowledge the diversity of ecological processes that might be captured by their greening maps and discuss plausible compositional, structural, phenological, physiological and trait‐mediated candidates based on a review of the literature. In the case of inference‐based attribution, hypotheses should be stated explicitly regarding the ecological processes underlying the gradients explored. If attribution is not possible, per‐pixel greening magnitude should be compared only across pixels that share a similar initial state, so that differences in trend magnitude reflect comparable ecological contexts. More fundamentally, approaches that invert the equifinality problem—by using spectral unmixing (and related methods) to track interpretable surface components rather than a single greenness index—may define the next generation of alpine vegetation change products and, ultimately, supersede greening maps as the primary lens for ecological interpretation.

These steps provide the necessary foundation for assessing drivers and consequences. Investigating the drivers of “greening” while leaving the underlying ecological processes unspecified is a precarious posture, because different processes may respond to different—and sometimes opposing—controls. For example, shrub encroachment, herbaceous densification, and treeline advance can all produce positive spectral trends, yet each may be governed by distinct combinations of temperature, snow regime, moisture, land‐use history, disturbance, and biotic interactions. Moreover, owing to intraspecific plasticity, the initial response of alpine vegetation to environmental change is often more likely to be physiological, phenological, or functional than compositional or structural (Bektaş et al. 2021, 2026; Henn et al. 2018). Consequently, over the course of the observational window, the dominant ecological process may shift within the same pixel, together with the controls that govern it. Collapsing these trajectories into a single greening metric risks conflating mechanisms and yielding correlations that are difficult to interpret mechanistically. Accordingly, methodological robustness and process attribution should precede driver inference: only once the radiometric signal is defensible and its ecological meaning is constrained does it become possible to attribute change to specific drivers and assess consequences for ecosystem functioning with confidence.

## 8 Next Steps in Satellite Remote Sensing of Alpine Vegetation Dynamics

Challenges ahead are less about identifying new corrections or inventing entirely new processing methods—many methodological issues have already been discussed in the literature—than about encouraging good practice in applying these corrections consistently and reporting unavoidable biases transparently. More substantially, the main challenge remains phenomenological: making ecological sense of spectral change and reducing equifinality through attribution. In that perspective, the most informative advances will likely come from study designs that explicitly bridge pixels and processes. While the last decade has seen a race toward ever larger extents and finer spatial resolution, progress in the coming decade should depend less on producing more greening maps than on reconnecting those maps to ground knowledge. This implies, at least in part, a return to well‐constrained study systems, in which satellite remote sensing is re‐anchored in calibration landscapes where pixels are ecologically informed, repeatedly observed, and interpretable in process terms. Approaches such as pixel‐walking, which reverse the usual logic by producing ground data specifically designed for remote‐sensing interpretation, provide a promising template for this shift by explicitly linking Landsat‐scale pixels to field‐observed ecological and environmental variation (Wong et al. 2024). Also, a key advantage of such well‐constrained study systems is that they make it possible to combine multiple sources of information across nested scales, which is critical for resolving phenomenological complexities.

In such calibration landscapes, UAV imagery, high‐resolution commercial satellites such as PlanetScope, and airborne hyperspectral or LiDAR acquisitions can all serve as intermediate layers between field observations and long‐term satellite archives. Sentinel‐2 already provides 10 m multispectral observations with a 5‐day revisit, and commercial constellations such as PlanetScope now offer near‐daily imagery at ~3 m, opening new opportunities to resolve alpine habitat mosaics at spatial grains that remain blurred in Landsat trends (Dixon et al. 2025; Yang et al. 2023). Extending this progression further, multispectral UAV observations have been used to map vegetation fractions or functional types at very high resolution and then aggregate this information to coarser satellite grids, helping constrain the ecological composition underlying medium‐resolution pixels (Chen et al. 2016; Morgan et al. 2020; Riihimäki et al. 2019). Airborne hyperspectral acquisitions already show how imaging spectroscopy can move beyond vegetation cover or broad functional types by resolving plant communities, biodiversity patterns, and trait‐related heterogeneity in mountain vegetation (Marcinkowska‐Ochtyra et al. 2019; Zhao et al. 2021). Although truly spaceborne applications remain fewer, recent studies using EnMAP and PRISMA suggest that easier access to hyperspectral data may, over the next decade, extend such approaches to species richness, forest diversity, and mountain grassland mapping at broader extents (Patriarca et al. 2025; Senf et al. 2025; Tagliabue et al. 2026). Forthcoming satellite missions are moving in the same direction, from denser multispectral acquisition with missions such as NASA's Landsat Next mission (Roy et al. 2025, 2026) to hyperspectral acquisition such as ESA's CHIME mission (Dufour et al. 2025). Finally, LiDAR acquisitions already provides critical information on vegetation structure which has been used to tighten the ecological interpretation of greening trends (Bayle et al. 2025; Bolton et al. 2018). Taken together, these developments illustrate multiple pathways for reconnecting pixels and processes, while also raising new challenges of cross‐mission integration, calibration, and interpretation. In that context, Earth Observation Foundation Models (EO‐FMs) may offer a useful way to assemble these heterogeneous datasets by learning shared representations from multi‐sensor archives, and thus help move from parallel, sensor‐specific signals toward more integrated observables that may better support ecological attribution (Brown et al. 2025).

However, the proliferation of new sensors and data streams will only translate into scientific progress if they are embedded in workflows that remain transparent, reproducible, and broadly accessible. This shift toward reproducible and scalable workflows has been made possible largely because free public Earth observation data—long exemplified by Landsat's 2008 open‐data policy and later reinforced by Copernicus—have progressively become the new normal in remote sensing (Wulder et al. 2022; Zhu et al. 2019), although this equilibrium remains fragile because it depends on continued political and institutional commitment rather than on any irreversible technical condition (Roy et al. 2025). A second, more recent layer of this transformation has come from cloud‐based platforms such as Google Earth Engine, which have greatly expanded access to satellite archives and planetary‐scale computation, lowering local hardware barriers and potentially broadening participation in remote‐sensing research, including in contexts with more limited computing infrastructure (Kumar and Mutanga 2018; Pham‐Duc et al. 2023; Vijayakumar et al. 2024). Beyond lowering computational barriers, cloud‐based platforms also facilitate the sharing and reuse of complete processing workflows. For example, the Landsat‐based greening trend workflow developed by Choler et al. (2025), itself inspired in part by procedures implemented in the LandsatTS R package (Berner et al. 2023) and shared as Google Earth Engine code, has already been reused in multiple alpine and mountain greening studies, illustrating how publicly accessible scripts can help harmonize trend estimation across regions (Gouda and Dubey 2026; Leng et al. 2026; Macek et al. 2025). Yet this progress is accompanied by new dependencies: major cloud infrastructures are operated by private companies, even when they host and process publicly funded datasets, and long‐term scientific autonomy should not rest on the continued availability of any single commercial platform. This dependency also suggests that the next norm in Earth observation should not be open data alone, but open data coupled with publicly governed cloud‐processing infrastructures, a direction that recent European initiatives such as the Copernicus Data Space Ecosystem are beginning to explore (Kovács et al. 2026). Finally, these questions of access and governance should not obscure a further constraint: the environmental cost of Earth observation itself. In the era of proliferating constellations and cloud computing, remote sensing data volumes are growing rapidly, and the storage, duplication, and processing of these archives already carry a measurable environmental footprint (Wilkinson et al. 2024). Multiplying sensors while lowering the barriers to large‐scale analysis will only intensify that pressure. The next step for satellite ecology should therefore not be more data by default, but more selective, interpretable, and energy‐aware uses of the data already collected.

## 9 Conclusion

Long satellite archives offer an unparalleled opportunity to document alpine vegetation change across entire mountain ranges and over the decades during which climate and land use have shifted most strongly. However, this paper argues that “greening” should not be treated as an ecological observation. A greening trend is the endpoint of a radiometric measurement and processing chain, and it remains ecologically ambiguous because multiple vegetation dynamics can generate similar spectral trajectories. The central message of this perspective is therefore that robust alpine inference requires addressing two intertwined layers of complexity in sequence. Methodological complexity must be tackled first, by maximizing the ground‐related fraction of the signal through bias‐aware workflows, explicit reporting of observation density, clear justification of any omitted corrections, and careful consideration of vegetation index choice. Establishing such a shared methodological baseline is essential for meaningful comparison across study sites and regions. Even when trends are methodologically robust, phenomenological complexity persists. Greening is equifinal, and its ecological meaning cannot be assumed from a vegetation index slope alone. Progress therefore depends on process attribution—using contextual priors as explicit hypotheses, validating trajectories with independent evidence where feasible, and increasingly developing process‐resolving observables that reduce reliance on one‐dimensional greenness. In that respect, spectral unmixing and related approaches offer a promising route toward component‐based products that are more directly interpretable in ecological terms, although their feasibility in alpine systems will depend on scale, calibration data, and the level of ecological detail targeted. This sequential logic matters because it conditions what can be learned about drivers and consequences. Driver analyses that ignore process diversity risk conflating mechanisms and producing correlations that are difficult to interpret mechanistically. Over the coming decade, the most informative advances will therefore come less from producing ever more detailed and larger‐area greening maps than from reconnecting satellite observations to ground knowledge through well‐constrained calibration landscapes, complementary observation systems across scales, and workflows that remain transparent, reproducible, and broadly accessible. In that sense, the next step for satellite ecology is not more data by default, but more selective, interpretable, and ground‐constrained uses of the increasingly diverse observations already available.

## Author Contributions

**Arthur Bayle:** conceptualization, investigation, methodology, writing – original draft, visualization, validation, formal analysis.

## Funding

This research has been supported by the Agence Nationale de la Recherche (project TOP, Trajectories of agrO‐Pastoral systems in mountains, grant no. ANR‐20‐CE32‐0002). This work received funding from the Zone Atelier Alpes project HERITAGE. LECA acknowledges the Agence Nationale de la Recherche (Grant nos. Labex OSUG@2020 and IA‐10‐LABX‐0056).

## Conflicts of Interest

The author declares no conflicts of interest.

## Data Availability

The alpine vegetation map of Niwot Ridge used in this study is available from https://doi.org/10.1080/00040851.1978.12003941. Code to compute the Simpson Diversity Index of alpine vegetation communities across a sequence of spatial resolutions, for Figure 3 panel b, is available at https://github.com/arthurbayle/perspective.
